# Peripheral Delivery of Neural Precursor Cells Ameliorates Parkinson’s Disease-Associated Pathology

**DOI:** 10.3390/cells8111359

**Published:** 2019-10-30

**Authors:** George Edwards, Nazaret Gamez, Enrique Armijo, Carlos Kramm, Rodrigo Morales, Kathleen Taylor-Presse, Paul E. Schulz, Claudio Soto, Ines Moreno-Gonzalez

**Affiliations:** 1 The Mitchell Center for Alzheimer’s Disease and Related Brain Disorders, Department of Neurology, The University of Texas Houston Health Science Center at Houston, 77030 Houston, TX, USA; George.A.Edwards@uth.tmc.edu (G.E.); Nazaret.GamezRuiz@uth.tmc.edu (N.G.); Enrique.Antonio.ArmijoFuentes@uth.tmc.edu (E.A.); Carlos.F.KrammBarria@uth.tmc.edu (C.K.); Rodrigo.MoralesLoyola@uth.tmc.edu (R.M.); Kathleen.B.TaylorPresse@uth.tmc.edu (K.T.-P.); Paul.E.Schulz@uth.tmc.edu (P.E.S.); Claudio.Soto@uth.tmc.edu (C.S.); 2 Dpto. Biologia Celular, Genetica y Fisiologia, Instituto de Investigacion Biomedica de Malaga-IBIMA, Facultad de Ciencias, Universidad de Malaga, 29010 Malaga, Spain; 3 Universidad de los Andes, Facultad de Medicina, Av. San Carlos de Apoquindo, 2200, Las Condes, 7620001 Santiago, Chile; 4 Centro Integrativo de Biología y Química Aplicada (CIBQA), Universidad Bernardo O’Higgins, 8370993 Santiago, Chile; 5 Networking Research Center on Neurodegenerative Diseases (CIBERNED), 29010 Malaga, Spain

**Keywords:** neuronal precursors, stem cells, Parkinson’s disease, therapy, intravenous, inflammation, clinical symptoms

## Abstract

Parkinson’s disease (PD) is a progressive neurodegenerative disorder characterized by loss of motor control due to a wide loss of dopaminergic neurons along the nigro-striatal pathway. Some of the mechanisms that contribute to this cell death are inflammation, oxidative stress, and misfolded alpha-synuclein-induced toxicity. Current treatments are effective at managing the early motor symptoms of the disease, but they become ineffective over time and lead to adverse effects. Previous research using intracerebral stem cell therapy for treatment of PD has provided promising results; however, this method is very invasive and is often associated with unacceptable side effects. In this study, we used an MPTP-injected mouse model of PD and intravenously administered neural precursors (NPs) obtained from mouse embryonic and mesenchymal stem cells. Clinical signs and neuropathology were assessed. Female mice treated with NPs had improved motor function and reduction in the neuroinflammatory response. In terms of safety, there were no tumorigenic formations or any detectable adverse effect after treatment. Our results suggest that peripheral administration of stem cell-derived NPs may be a promising and safe therapy for the recovery of impaired motor function and amelioration of brain pathology in PD.

## 1. Introduction

Parkinson’s disease (PD) is the second most common neurodegenerative disease. It is characterized by the loss of dopaminergic neurons of the substantia nigra pars compacta (SNpc) leading to a progressive movement impairment that can include tremors, bradykinesia, rigidity, and postural instability [1,2]. PD patients also display non-motor symptoms such as anxiety and depression, psychosis, gastrointestinal problems, and sleep dysregulation [3,4,5]. The pathophysiology underlying PD includes the aggregation of misfolded alpha-synuclein protein that generates intracellular Lewy bodies [2,6,7]. In addition, there is an associated neuroinflammatory process governed by the action of microglia and astrocytes, which has been postulated to play a crucial role in PD pathogenesis [8,9]. The presence of reactive microglia and an elevation in the number of astrocytes have been detected in the SNpc of PD patients and in mouse and primate models of PD systemically injected with 1-methyl-4-phenil-1,2,3,6-tetrahydropyridine (MPTP) [10,11,12,13].

Some treatments, such as deep-brain stimulation and pharmacotherapy, have shown benefit in reducing the symptoms, but there is no cure for PD [14,15,16]. Stem cell (SC) therapy using neuronal transplantation has shown some promise in combating PD [17,18,19]. Embryonic stem cells (ESCs) are pluripotent cells derived from the inner mass of the blastocyst. Although promising, their use in research and for therapeutic purposes has generated ethical concerns. Mesenchymal stem cells (MSCs) are multipotent stromal cells that can be obtained from the individual’s bone marrow and have the ability to differentiate into a variety of cells, such as connective tissue cells (muscle, fat, bone, and cartilage) [20,21]. MSCs offer advantages over ESCs, such as being derived from the patient to whom they are given, a low risk of tumor formation, no risk of an immune response, and no ethical dilemmas. Both ESCs and MSCs, are capable of generating neural precursors (NPs), which are a promising therapeutic strategy for neurological disorders due to their capacity to differentiate into functional astrocytes, oligodendrocytes, and mature neurons. Their genetic stability, limited propagation, lack of tumorigenicity, and their circumvention of ethical concerns make them ideal tools for therapeutic interventions [22].

Previous studies have shown that MSCs and dopaminergic neurons derived from human pluripotent stem cells can ameliorate PD symptoms when administered directly into the brain of PD models [23,24]. Although the mechanism is not well understood, it is known that MSCs can produce neurotrophic factors, which could facilitate cell survival, mitigate oxidative stress, increase the production of anti-inflammatory cytokines, inhibit glial activation, and suppress cellular apoptosis [25,26,27].

Clinical trials in humans have tested the safety and efficacy of intracerebral transplantation of human ESCs-derived NPs into the striatum in PD patients. Nevertheless, attempts to get stem cells into the brain to replace the cells lost in neurodegenerative diseases, such as PD, have been unsuccessful. In addition, post-mortem studies in brains of treated patients show presence of very few stem cells with no evidence that they differentiated into cells that replaced the lost ones. In addition, surviving cells contained Lewy bodies [28,29].

Studies performed using an intravenous (i.v.) infusion of stem cells in stroke patients, however, showed that it is not necessary to deliver stem cells into the brain to have a positive effect on the clinical outcome [30]. Furthermore, intravascular infusion techniques are safe, less invasive than brain grafting, and could constitute a more convenient administration approach for stem cell therapy in the clinic. Here, we investigate the effect of the peripheral inoculation of NPs, derived from ESCs and MSCs, as a non-invasive therapy to ameliorate the motor symptoms and modulate the disease-associated neuroinflammation in a drug-induced (MPTP) mouse model of PD. Safety, in terms of tumor formation, was also evaluated.

## 2. Results

### 2.1. Generation of Neural Precursors for Cell Therapy

**Figure 1:**
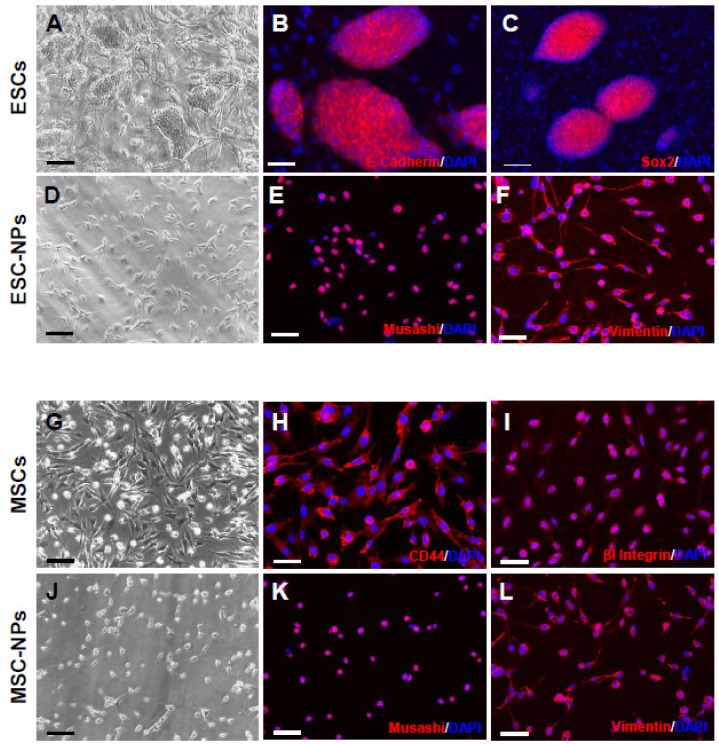
Neural precursors obtained from embryonic and mesenchymal stem cells. Morphology of ESCs at passage number 10 (**A**). Immunocytochemistry for E-cadherin (**B**) and SOX2 (**C**). NPs have elongated bipolar morphology, end feet, and oval nuclei (**D**). Immunocytochemistry for the NP markers musashi (**E**) and vimentin (**F**). Morphology of MSCs at passage number 7 (**G**). Immunocytochemistry for CD44 (**H**) and βI integrin (**I**). NPs showed similar morphology as we observed on ESC-NPs (**J**). MSC-NPs were positive for musashi (**K**) and vimentin (**L**). Nuclei were stained with DAPI (blue). Scale bar A, D, G, J: 25 μm; B, C, E, F, H, I, K, L: 50 μm.

To develop a systemic, stem cell-based treatment for PD, we first produced NPs from both mouse ESCs and MSCs (Figure 1A,G). ESCs were characterized using the pluripotency markers E-cadherin and Sox2 (Figure 1B,C), whereas MSCs were positive for the markers CD44 and βI integrin (Figure 1H,I). NPs can be derived in vitro under the influence of mitogens, such as EGF and FGF-2, and they retain the potential to differentiate into neurons, astrocytes, and oligodendrocytes [31,32,33]. ESCs and MSCs were subjected to a multistep protocol for converting them into a monolayer of self-renewing NPs. ESCs and MSCs were adapted for at least three passages on gelatin in order to expose them to neural induction stimuli. These cells, designated as ESC-NPs and MSC-NPs, were continuously and homogenously propagated, with no significant differences in morphology or growth rate for over 30 passages. Generated NPs showed elongated bipolar morphology, end feet, and oval nuclei (Figure 1D,J). NPs maintained in self-renewing conditions were positive for markers typically observed in NPs by immunocytochemistry, including musashi (Figure 1E,K) and vimentin (Figure 1F,L).

### 2.2. Short-Term Attenuation of PD-Induced Motor Deficits After Stem Cell Therapy

**Figure 2:**
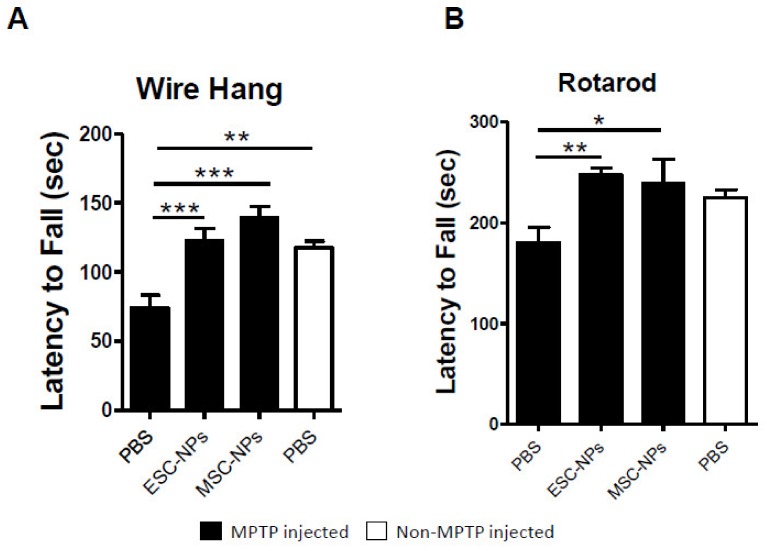
Behavioral assessment of MPTP-induced mice 10 days after NP treatment. Bar graphs reveal the latency to fall in seconds (sec) in the wire hang (**A**) and rotarod (**B**) tests. In both tests, we analyzed MPTP-induced animals (black) inoculated with ESC-NPs, MSC-NPs, and PBS 10 days after the last injection, and age matched non-MPTP-injected mice (white) treated with PBS. Data are expressed as mean ± s.e.m. ANOVA, Dunnett’s multiple comparison test, * *p* < 0.05, ** *p* < 0.01, *** *p* < 0.001. N = 9–10 mice/group.

To test the efficacy of an i.v. injection of NPs to treat PD pathology, we used MPTP-treated mice. This is perhaps the most widely used model of PD, which is characterized by loss of dopaminergic neurons in the nigrostriatal pathway with accompanying Parkinsonism. C57BL/6J mice were treated intraperitoneally with MPTP hydrochloride (30 mg/kg) administered daily for 5 consecutive days to generate the PD-like phenotype. These MPTP mice demonstrated impairment in the motor specific tasks of wire hanging and rotarod as compared to non-MPTP injected mice by student t-test (*p* < 0.001 and *p* < 0.05, respectively) (Figure S1). Ten days after the last MPTP injection, mice were inoculated twice over a week with 1 × 10^6^ cells of either ESC-NPs or MSC-NPs by tail vein injection. Animals injected with PBS were used as controls. Ten days after the last cell injection, mouse groups were behaviorally assessed by the wire hang and rotarod tests to determine the effect of the inoculation in clinical signs (Figure 2).

ESC-NP- and MSC-NP-inoculated mice revealed a better wire hanging performance compared to age-matched PBS-injected mice and displayed better grip strength and motor coordination by Dunnett’s multiple comparison test (*p* < 0.0001 and *p* < 0.0001, respectively), as shown in Figure 2A. One-way ANOVA demonstrated an overall effect among the groups by wire hang and rotarod tests (*p* < 0.0001 and *p* < 0.05, respectively). Moreover, the rotarod test (Figure 2B) showed a significant difference in the latency to fall between ESC-NPs and MSC-NPs compared to PBS-injected mice (*p* < 0.01 and *p* < 0.05, respectively). The level of performance was similar to animals not treated with MPTP as observed by no main differences in behavior between the control non-MPTP mice treated with PBS and the MPTP-injected mice treated with NPs. Therefore, NP peripheral inoculation induced amelioration of clinical symptoms observed in MPTP-exposed mice 10 days after treatment.

**Figure 3:**
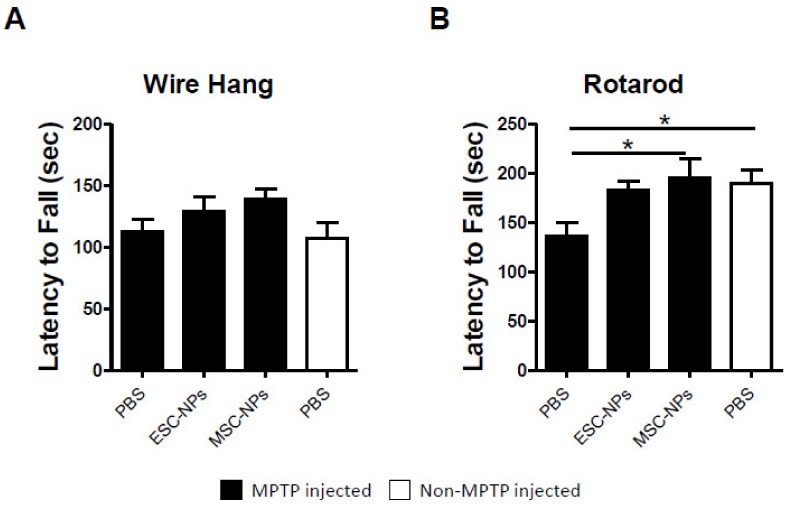
Behavioral assessment 3 months after NP treatment. Bar graphs represent wire hang (**A**) and rotarod (**B**) performance represented as the mean latency to fall (seconds, sec). MPTP-injected mice (black) were treated with ESC-NPs, MSC-NPs, and PBS 3 months after the last injection with NPs. Age matched non-MPTP-injected mice (white) treated with PBS were used as a control. Data are expressed as mean ± s.e.m. ANOVA, Dunnett’s multiple comparison test, * *p* < 0.05. N = 9–10 mice/group.

In an effort to determine the long-lasting effects of the NP peripheral treatment, another set of animals was subjected to the same experimental conditions but sacrificed 3 months after the NP inoculation. Figure 3A illustrates their behavioral performance in the wire hanging test. We observed that there was no significant difference in latency to fall from the wire between ESC-NPs or MSC-NPs and PBS-inoculated mice (one-way ANOVA, *p* > 0.05). However, there was also no difference between animals injected with MPTP and untreated wild type mice, indicating that at this time, the deleterious effect of MPTP was not detectable by the wire hanging test. Conversely, there was a significant difference among the groups (one-way ANOVA, *p* < 0.05), where motor performance on the rotarod (Figure 3B) was significantly better in the MSC-NP mice and non-MPTP PBS mice, than the PBS-injected mice (*p* < 0.05).

These results indicate that NPs from ESC and MSC origin have an early beneficial effect on motor symptoms in motor-dependent behavior paradigms. Because the MPTP-induced model does not present a progressive pathology, behavioral data demonstrate that the treatment is less robust over time but still significant.

### 2.3. NP Therapy Does Not Increase Dopamine Levels but Modulates Neuroinflammation

MPTP gets into CNS efficiently and induces the loss of dopaminergic neurons. These mice display reduced levels of tyrosine hydroxylase (TH) expression, a measurement of dopamine levels, in the SNpc compared to non-injected mice. To determine whether infusion of NPs modified levels of dopaminergic neurons, we analyzed the immunoreactivity (IR) of TH in NP and PBS-treated animals. Histological analysis of TH-IR demonstrated no differences in the levels of TH in the experimental and control groups at short (10 days after treatment) and long (3 months after treatment) terms, suggesting that the changes observed in the motor tests were not due to modifications in the dopamine levels (Figure S2). Therefore, we focused our study on whether other hallmarks of PD pathology were influenced by the peripheral inoculation of NPs.

**Figure 4:**
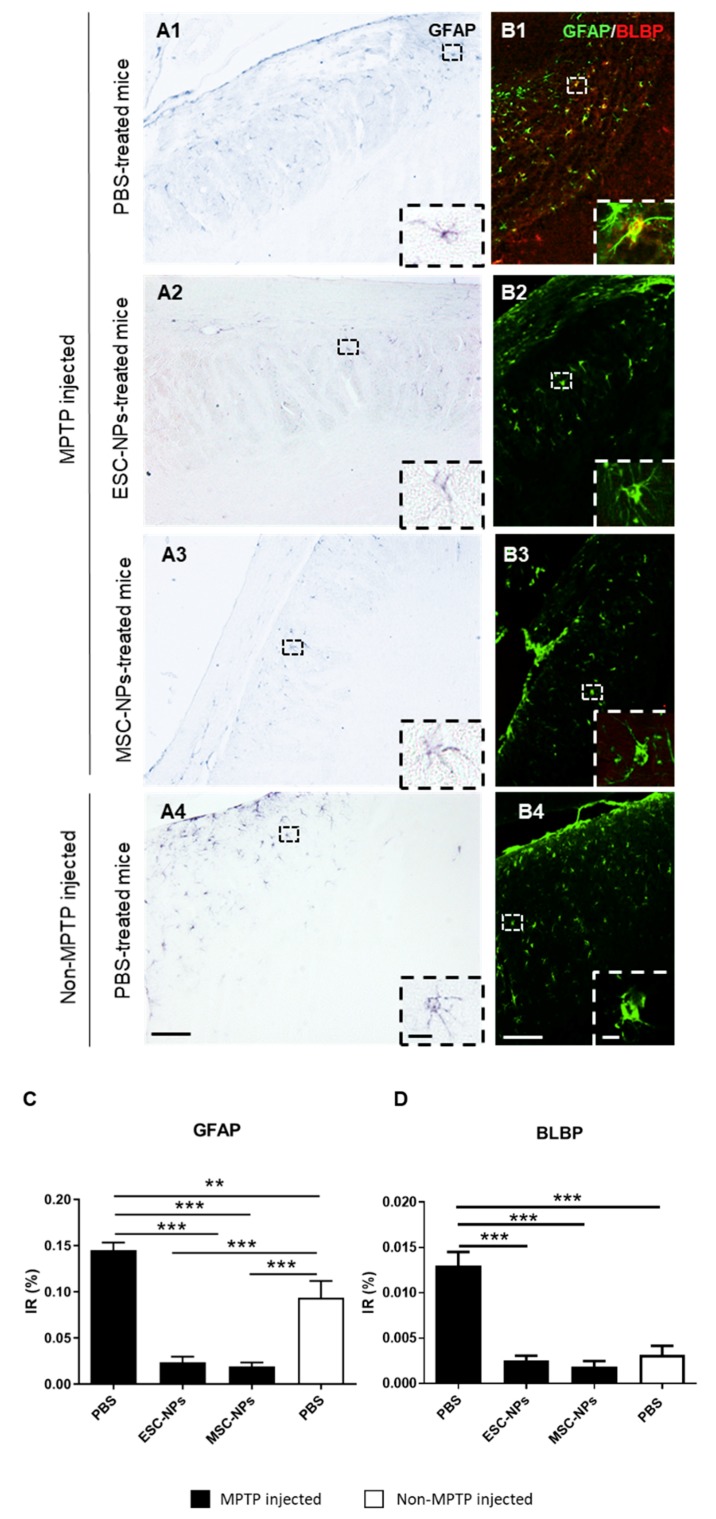
MPTP-injected mice show reduced astrogliosis after NP peripheral treatment. MPTP inoculated ESC-NPs and MSC-NPs and PBS-treated mice, along with non-MPTP PBS mice, were sacrificed 10 days after the infusion. Brains were collected and astrocytosis was evaluated by histology. Representative images for bright field GFAP immunostaining (**A1**–**A4**) and double immunofluorescence for GFAP and BLBP (**B1**–**B4**) in the striatum of MPTP-injected and non-MPTP-injected age-matched mice are presented. Insets show details of immunopositive astrocytes depicted in dashed squares. Image analysis quantification of the GFAP (**C**) and GFAP/BLBP-positive cells (**D**) in the whole brain (ANOVA, ** *p* < 0.01; ***p < 0.001). Scale bar: 100μm (A1–A4, B1–B4); 10μm (insets). N = 6 mice/group, 8 sections/mouse.

**Figure 5:**
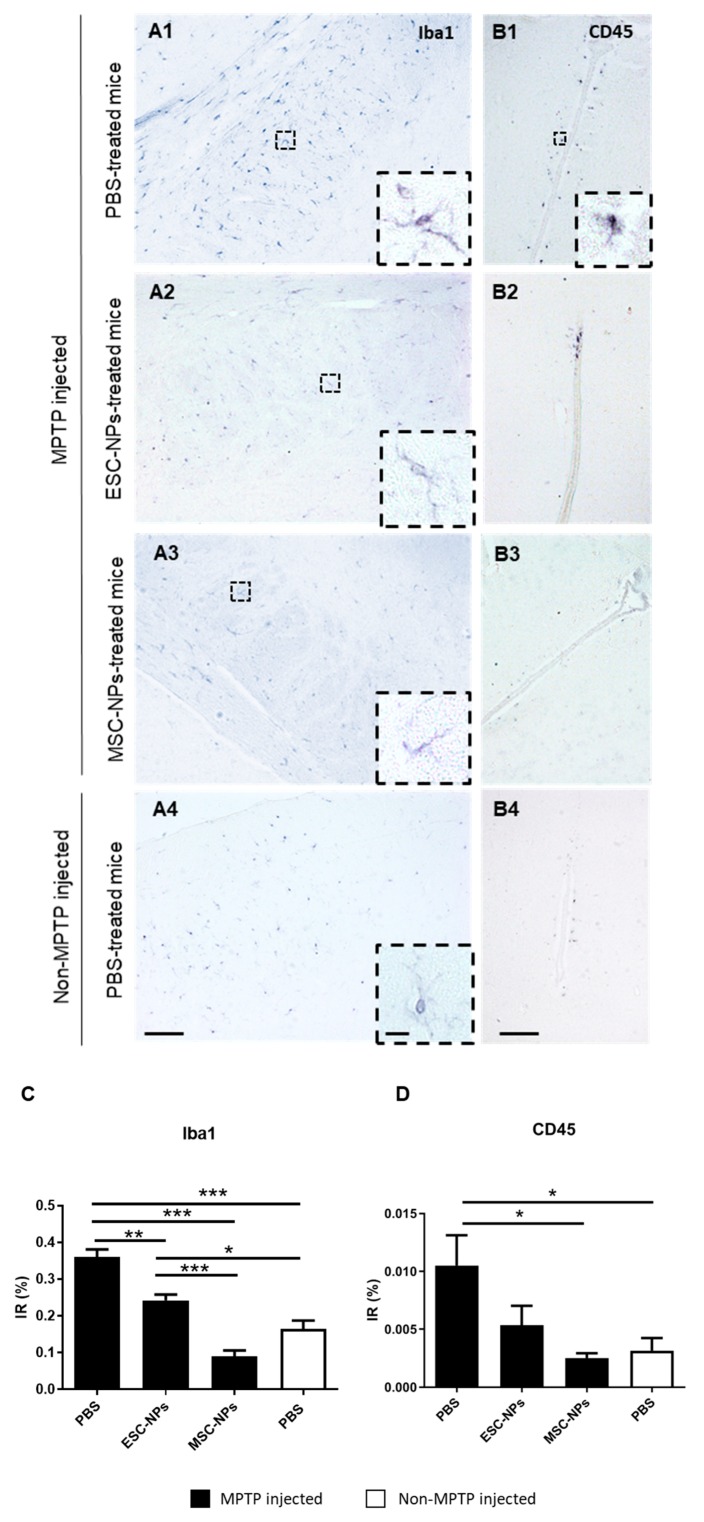
NPs treatment reduces early microglial and macrophage load in PD mice. MPTP-injected mice were evaluated 10 days after treatment with PBS, ESC-NPs and MSC-NPs. Representative bright field microphotography in the striatum (**A**) and third ventricle (**B**) are shown. Immunostaining against Iba1 for microglial cells (A1–A4) and CD45 for macrophages (B1–B4) was performed in 8 brain sections throughout the brain. Insets show details of Iba1 and CD45 positive cells depicted in dashed squares. Image analysis quantification of Iba1 (**C**) in the brain of MPTP-injected animals treated with either ESC-NPs, MSC-NPs, PBS, or non-MPTP PBS injected controls (ANOVA, ** *p* < 0.01, *** *p* < 0.001). Immunoreactive (IR) quantification of monocyte-derived macrophages (**D**) of all groups (ANOVA, * *p* < 0.05). Scale bar: 100 μm (A1–A4, B1–B4); 10 μm (insets). N = 6 mice/group, 8 sections/mouse.

A chronic inflammatory response is thought to contribute to the pathogenic processes underlying PD. For that reason, we next tested the effects of stem cells on neuroinflammation. To investigate the effects of NPs on astrogliosis and microgliosis in the MPTP-induced PD model, immunohistochemistry analysis and quantification was performed for inflammatory markers throughout the brain. The non-MPTP PBS injected group was also subjected to neuroinflammation analysis (Figure 4A). We found a significant difference among the groups in GFAP and BLBP-expressing astrocyte levels in the brain (one-way ANOVA, *p* < 0.0001). MPTP-injected mice inoculated with PBS displayed significant astrogliosis compared to the non-MPTP PBS control group (*p* < 0.01). Treatment with both ESC- and MSC-NPs significantly decreased GFAP expression in MPTP-injected mice when compared to the PBS control group and to the non-MPTP injected group (*p* < 0.001) (Figure 4C). Then, immunostaining of reactive astroglia was performed using the BLBP (brain lipid-binding protein) antibody. BLBP is a marker of radial glial and reactive astrocytes as well as adult neural stem cells [34]. We quantified cells that were positive for both GFAP (in green) and BLBP (in red) to determine the amount of reactive astrocytes. Histological analysis demonstrated that BLBP-expressing astroglial cells were reduced in MPTP-injected mice receiving the ESC-NP (*p* < 0.001) and MSC-NP (*p* < 0.001) inoculation relative to PBS-injected animals (Figure 4B). Furthermore, BLBP-positive astrocyte levels in NP-treated mice were similar to non-MPTP PBS control mice. There was a significant difference among the evaluated groups by one-way ANOVA (*p* < 0.0001) (Figure 4D). For the expression of Iba-1, a microglia marker. MPTP induced a significant increase in Iba-1 burden with respect to non-MPTP injected mice (Figure 5A1–4). The i.v. treatment using both ESC- and MSC-derived NPs significantly reduced the burden of Iba1-immunopositive microglia compared to the PBS injected mice (Figure 5C). Interestingly, the reduction in reactive microglia was more prominent in animals treated with NPs derived from MSCs when compared to ESCs (*p* < 0.001), while non-MPTP PBS animals had less Iba-1 IR compared to ESC-NPs mice (one-way ANOVA, *p* < 0.05). Monocytes can infiltrate the brain and differentiate into macrophages. To determine whether the reduction of Iba1 was due to a decrease in microglial cells (Iba1/CD45^lo^) or monocyte/macrophage (Iba1/CD45^hi^) infiltration, we immunostained brain sections using the CD45 antibody [35]. The CD45 signal was shown mostly in animals treated with MPTP without treatment of NPs (Figure 5B1–4). The representative pictures and immunoreactivity (IR) quantification data demonstrate that the levels of CD45 were significantly reduced in animals treated with MSC-NPs (Figure 5D), showing that there is a decrease in monocyte/macrophage infiltration. CD45-positive cells were found mainly around the third ventricle and a significant difference between the groups was found (one-way ANOVA, *p* = 0.014).

### 2.4. Intravenous Treatment with NPs is Not Tumorigenic

**Figure 6:**
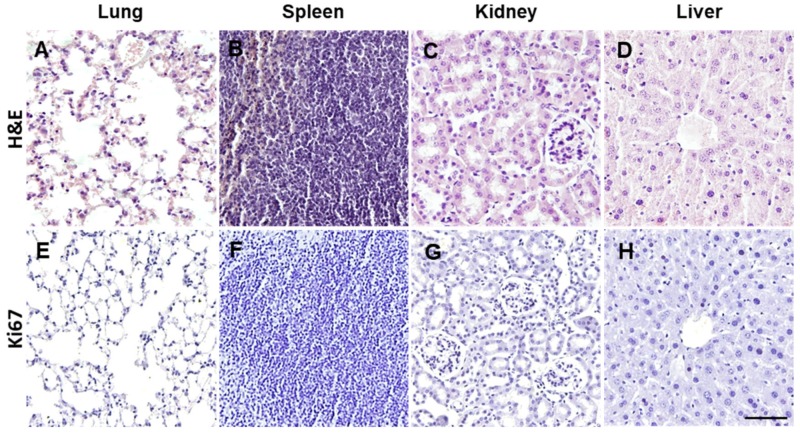
Tumorigenicity assessment of NP treatment. ESC-NPs and MSC-NPs infused animals were assessed for tumor formation 10 days, 3 months, and 9 months after the injection. Representative microphotographs of histological analysis using general hematoxylin-eosin staining (**A**–**D**) and immunohistochemistry for Ki67 counterstained with hematoxylin (**E**–**H**). Representative pictures of lung (**A**), spleen (**B**), kidney (**C**), and liver (**D**) were absent of any tumorigenic formation. Scale bar: 50 μm. N = 8 mice/group.

In order to test the safety of the i.v. inoculation with NPs, we carefully checked for the development of tumors from injected NPs by palpation and visual screening once every 2 weeks after the last inoculation. No tumor formation was observed during the execution of these experiments. In addition, hematoxylin-eosin general staining was performed in the brain, lung, spleen, kidney, and liver to determine the presence of tumor formation in animals sacrificed 10 days, 3 months, and 9 months after the treatment. No evidence for tumor cell masses was detected in any of the animals analyzed, as observed in the representative pictures in Figure 6A–D. We also analyzed the tissue samples using the proliferation antibody Ki67, and no tumor formation was found (Figure 6E–H).

## 3. Discussion

PD is a devastating neurodegenerative disease associated with loss of dopaminergic neurons in the striatum, brain inflammation and accumulation of α-synuclein aggregates in the form of Lewy bodies [1]. Currently, there is no cure for this disease and available treatments only mitigate the symptoms [36]. One potential therapy that has gained support involves the use of stem cells. The goal of this study was to test whether the peripheral injection of NPs obtained from ESCs and MSCs could safely ameliorate the motor abnormalities and associated neuropathology in a mouse model of PD.

To this end, NPs obtained from embryonic and mesenchymal stem cells were inoculated into MPTP-induced mice. We found that animals treated with both cell types showed an amelioration in their behavioral impairment compared to PBS-treated control animals in the rotarod and wire hanging tasks as early as 10 days after stem cell therapy. Importantly, motor-related benefits somewhat persisted 3 months following treatment. Chiefly, MSC-NP treated mice had a restored ability to stay on the rotarod compared to PBS-inoculated mice, indicating an amelioration of the motor symptoms of MPTP-induced PD, while ESC-NP-treated rodents presented a trend in improvement, as well. Furthermore, 3 months after ESC-NP and MSC-NP therapy by i.v. injection, experimental mice demonstrated improved motor capacities, though less robust compared to the 10 day post-treatment time period, compared to PBS-treated mice.

This study was performed in 2-month-old female C57BL/6J mice. Gender could play an important role in the clinical treatment and ultimate outcome of PD. In fact, clinical data has reported a slight prevalence and incidence in males and quicker onset of PD. On the other hand, females tend to develop tremor-dominant PD [37]. Thus, further evaluation of the gender-specific effect of this therapy needs to be determined.

Previous reports using SC therapy by intracerebral injection noted that the introduced cells were able to survive and were present in brain-yielding novel neurons, as evaluated by the increased expression of TH in PD brains compared to controls [23,38,39,40]. Survival was very low; however, it remains unclear whether the injected cells were synaptically integrated and whether the changes observed were due to replacement of lost neurons by these remaining cells. Another alternative is that the cells release SC-derived factors that enhance endogenous cells for support. The amelioration of clinical symptoms observed in our cell-treated MPTP-induced mice was not due to an increase in TH levels after the treatment, suggesting an alternative mechanism of action for the injected cells. Thus, other factors associated with the MPTP treatment may be improved after NP treatment leading to an amelioration of PD clinical manifestations.

As a better performance in behavioral tasks was observed 10 days after the treatment with NPs, the brain of these mice were analyzed for potential mechanisms of action. Currently, there are two proposed mechanisms responsible for neuronal cell death in PD and other neurodegenerative disorders. The “cell-autonomous” mechanism refers to an accumulation of intrinsic damage in the degenerating neurons, whereas the “non-cell-autonomous” mechanism results from an indirect degeneration of the affected neurons caused by pathological interactions with neighboring cells, mostly resident glial cells (microglia and astroglia). These glial cells govern the inflammatory process that has been repeatedly linked to neurodegeneration in PD, but whether the neuroinflammation is a cause or a consequence of neuronal degeneration remains unanswered [8].

The MPTP model for PD is known to generate excessive cellular reactive oxygen species (ROS), which induce oxidative stress and neuroinflammation linked to degeneration of nigrostriatal DA neurons [41]. In fact, neuroinflammation is suggested as the cause and the effector of the neurodegenerative process in PD animal models. A growing body of evidence has suggested that higher glial cell activation is connected to DA neuronal loss in the SNpc [42]. The present study demonstrated that intravascular infusion of both ESCs-NPs and MSCs-NPs in MPTP-induced PD models significantly reduced the burden of reactive astroglia and microglia cells throughout the brain compared to the PBS-injected mice 10 d.p.i. Even more, the activation of astroglia and macrophages was drastically decreased in the MPTP mouse model after the i.v. treatment with NPs, indicating a potential mechanism of amelioration of motor impairment in treated animals. More studies need to be done to determine the direct effect of infused NPs on glial activation and the potential effect on motor behaviors and neurodegenerative diseases.

Another viable hypothesis is that SC-induced release of trophic factors could contribute to their efficacy. It has been suggested that excretion of certain trophic and growth factors, such as brain-derived neurotrophic factor (BDNF), glial-derived neurotrophic factor (GDNF), stromal cell-derived factor (SDF), and transforming growth factor-β (TGF-β), provide benefit following SC treatment in injury and disease models. GDNF delivery in PD animal models and patients has had some encouraging results [43,44].

Therefore, peripheral delivery of NPs could be considered as a therapeutic strategy to ameliorate PD symptoms using a non-invasive approach with no detectable side effects. For instance, NPs can be derived from MSC present in the adipose tissue from the same individual to avoid any immune rejection. In addition, the use of ESC-NPs would involve ethical issues, and their effect seems to be lower than that obtained from MSC-derived NPs in our model. An alternative therapy could be the use of NPs from induced pluripotent SCs (iPSCs) that can be obtained from the patient´s isolated fibroblasts. The similarity between mouse and human NPs also remains to be evaluated and if those obtained from humans have the ability to produce the same derived factors able to reduce the inflammatory process. Transcriptomic and proteomic analyses of both mouse and human NPs are currently ongoing in our lab to determine the potential translation of this therapy into humans. Another therapeutic approach could involve the use of conditioned media obtained from NPs in culture in an effort to enrich the inoculum with the released factors in a cell-free treatment, but it could well be that factors produced in vivo and responsible for the observed effects are not similar to the ones released in vitro.

The present study suggests that the intravascular infusion of NPs obtained from both ESC and MSC is an innocuous, non-invasive treatment—as compared to intracerebral injection of SCs—that may be used as a treatment for PD. Effects seen here could be mostly due to the modulation of the neuroinflammatory response and amelioration of motor impairment without tumorigenicity. As brain inflammation is commonly involved in many other neurodegenerative diseases, the present strategy could be a therapeutic candidate for Alzheimer’s disease, multiple sclerosis, amyotrophic lateral sclerosis, and Huntington’s disease, among others.

## 4. Materials and Methods

### 4.1. Preparation of Mouse NPs from ESC and MSCs

Mouse embryonic stem cells (ESC; derived from a 129sv mouse) were kindly provided by Dr. Eva Zsigmond (Director of Transgenic and Stem Cell Service Unit at the Brown Foundation Institute of Molecular Medicine for the Prevention of Human Diseases). ESCs were plated on irradiated mouse embryonic fibroblasts cells CF-1 (iMEFs; MTI-GlobalStem, Rockville, MD, USA) and cultured in mouse ESC medium (DMEM (Invitrogen, Carlsbad, CA, USA), 15% FBS (Atlanta Biologicals, Flowery Branch, GA, USA), 1× nonessential amino acids (Invitrogen), 1× Glutamax (Invitrogen), 1mM sodium pyruvate (Invitrogen), 10 mM HEPES (Invitrogen), 1000 U/mL leukemia inhibitory factor (LIF) (R&D Systems, Minneapolis, MN, USA), 0.1% 1-thioglycerol (Sigma, St. Louis, MO, USA) and 1× antibiotic and antimitotic stock solution (Invitrogen). The culture medium was replaced daily. Mouse mesenchymal stem cells (MSC; derived from a C57BL/6 mouse, Cyagen, Santa Clara, CA, USA) were cultured using the mouse mesenchymal stem cell growth medium (Cyagen). The culture medium was replaced every three days. Derivation of neuropotent self-renewing NPs was performed as previously described [45]. ESC and MSCs were harvested, resuspended in neural induction medium (DMEM/F12 and Neurobasal medium (1:1, Invitrogen), 0.5× N2 supplement (Invitrogen), 1× B27 supplement (Invitrogen) and 1× antibiotic and antimitotic stock solution (Invitrogen) and transferred to 0.1% gelatin-coated plates. Culture medium was replaced every day. After 12 days, cells were harvested by Accutase (Millipore, Burlington, MA, USA), resuspended in neural progenitor expansion media (DMEM/F12, 1% N2 supplement, 20 ng/mL EGF and 20 ng/mL bFGF) (R&D Systems) and 1× antibiotic and antimitotic stock solution and transferred to Geltrex LDEV-free reduced growth factor basement membrane matrix (1:100, Invitrogen)-treated dishes. Medium was changed as needed.

### 4.2. Immunocytochemistry

ESCs and MSCs were fixed with Bouin’s solution (Sigma-Aldrich, St. Louis, MO, USA) for 15 min at 4 °C and stored. Fixed cells were blocked, permeabilized and incubated with the primary antibodies overnight in TCT buffer (0.25% Carrageenan and 0.1% Tween-20 in Tris-HCl buffer pH 7.8). The primary antibodies used were: Sox2 (1:100, Abcam, Cambridge, UK), E-Cadherin (1:15, BD Biosciences, San Jose, CA, USA), CD44 (1:100, Abcam), βI Integrin (1:100, Abcam), Musashi (1:200, Abcam) and Vimentin (1:100, Abcam). After rinsing, appropriate secondary antibodies conjugated to Alexa fluorophores 488 or 598 (Molecular Probes, Invitrogen, Carlsbad, CA, USA) were diluted at 1:500 in TCT solution and incubated for 1 hr at room temperature. After rinsing, cells were mounted in Vectashield hardset mounting medium with 4’,6-diamidino-2-phenylindole (DAPI) (Vector laboratories) to counterstain nuclei. Images were acquired with a Leica DMI 6000B microscope.

### 4.3. Induction of Parkinson’s Disease Phenotype

As a model of PD, we used the well-established and widely used mouse PD model based on the administration of MPTP (1-methyl-4-phenyl-1,2,3,6-tetrahydropyridine). Two-month-old female C57BL/6J mice (Jackson Laboratories, Bar Harbor, ME, USA) were treated intraperitoneally with MPTP hydrochloride (30 mg/kg) administered daily for 5 consecutive days, as previously reported, to induce selective destruction of dopaminergic neurons of the nigrostriatal tract [46]. The cumulative dose was 150 mg/kg and control mice received the same volume of PBS vehicle. Immediate Parkinsonism traits were perceived following MPTP induction. C57BL/6J animals were housed in groups of up to five in individually ventilated cages under standard conditions (22 °C, 12 h light–dark cycle) receiving food and water ad libitum. All animal experiments were carried out in accordance with UTHealth IACUC regulations (AWC-14-0094 and AWC-17-0140).

### 4.4. NP Treatment

Following a recovery period of 10 days after MPTP injection, experimental mice were inoculated with 1 × 10^6^ cell count (300 μL) of either ESC or MSC differentiated into mouse NPs or PBS for controls by tail vein injection. Inoculation was done twice in a one-week span. ESC-NPs, MSC-NPs, and PBS mouse groups were behaviorally assessed before sacrifice at 10 days, 3 months, and 9 months after treatment. Animals were sacrificed by CO_2_ inhalation, perfused with PBS-EDTA, and their brains, lungs, liver, kidneys, spleen, and pancreas removed, post-fixed into formalin solution, and embedded in paraffin for histological analysis. Animals were visually inspected for tumor formation. Tissues were visually analyzed for any anomaly, including changes in size, color or shape of the organ and existence of tumorigenic masses.

### 4.5. Wire Hang

Motor coordination and strength was measured by the wire hang task as previously described [47]. Briefly, a 40-cm long 2-mm thick metallic wire was placed 38 cm above a clean home-cage. The animal was placed in the middle of the wire with all four limbs and gently turned upside-down along the axis of the wire. The time until the mouse completely releases its grasp and falls was recorded in 3 minutes using 3 trials. The animal had a 30 second recovery interval until the next trial. The animal was prevented from balancing and limited the use of the tail by the observer.

### 4.6. Rotarod

Motor coordination and activity, grip-strength, and fatigue are all measurements that are assessed by the commonly used behavioral task of rotarod (Med Associates Inc., Fairfax, VT, USA). As previously described [48], the animal was placed on a horizontal oriented, rotating cylinder that accelerated from 4 to 40 rpms over a 5 minute trial. The latency to fall and best trial were quantified over 3 trials with 2 minutes interval rest. Six minutes was used as the final cut-off latency to fall measurement. In addition, one difficulty of the task is the rodent’s desire to grip the rotating rod to prevent falling; thus, 2 consecutive rotations of the animal clinging was considered as a latency to fall measurement. The animals were aided for 30 seconds in the first trial to prevent unnecessary behavior (i.e., turning and false-positive falls/jumps).

### 4.7. Immunohistochemistry

Paraffin coronal sections of 10 µm thickness were first treated with 3% H_2_O_2_–10% v/v methanol in PBS at a pH of 7.4 for 20 min to inhibit endogenous peroxidase. For general antigen retrieval, sections were previously heated at 80 °C for 30 min in 10 mM citrate buffer at pH 6.0 and were incubated with the primary antibody overnight at room temperature. The following primary antibodies were used for this study: rabbit polyclonal anti-tyrosine hydroxylase (1:1000; Millipore Sigma), rabbit polyclonal anti-Iba1 (1:1000; Wako Chemicals, Richmond, VA, USA), mouse monoclonal anti-GFAP (1:1000; Millipore Sigma), rabbit polyclonal anti-BLBP (1:1000; Abcam), rabbit polyclonal anti-CD45 (1:1000; Abcam), and goat polyclonal anti-Ki67 (1:200, Santa Cruz Biotechnology, Dallas, TX, USA). The tissue-bound primary antibody was detected by the incubation with the corresponding HRP-linked secondary antibody (1:500; Vector Laboratories) for 1.5 hr and visualized using diaminobenzidine (DAB) substrate. Samples were dehydrated in graded alcohol, cleared in xylene, and covered with DPX for bright field analysis. For double immunofluorescence labeling, sections were first sequentially incubated with the indicated primary antibodies (GFAP and BLBP) followed by the corresponding Alexa 488/594 secondary antibodies (1:500; Invitrogen). Sections were then rinsed and covered with Vectashield hardset mounting medium with DAPI (Vector laboratories) for immunofluorescent evaluation.

### 4.8. Quantitative Image Analysis

Glial burden was defined as the percentage of area stained with anti-GFAP (astrocytes), anti-BLBP (reactive astrocytes), anti-Iba1 (microglia) and anti-CD45 (macrophages) by the total area analyzed, as previously described [49,50]. Six to eight sections per mouse were immunostained and examined under the microscope (DMI6000B, Leica). Digital images were acquired with the program Surveyor and were analyzed using ImageJ version 1.43 software (NIH). The GFAP, Iba1, CD45 and double GFAP/BLBP immunopositive signal in the brain was converted into 8-bit gray scale and their intensity was thresholded to quantify the immunoreactivity (IR) area per total area analyzed. TH optical density (OD) was determined as the intensity of the immunoreactive signal (0–255) divided by the area analyzed (arbitrary units). The TH-IR area was defined as the area stained with anti-TH antibody in pixel units (px^2^).

### 4.9. Statistical Analysis

Graphs are expressed as means ± standard error of the mean (s.e.m.). After confirming normal distribution with Skewness and Kurtosis statistic tests, student t-test or one-way analysis of variance (ANOVA), followed by a post-hoc Dunnett’s multiple comparison or Tukey’s tests, were used to analyze differences among groups. Statistical differences for all tests were considered significant at the confidence level of p < 0.05. Statistical analysis was performed using GraphPad Prism 5.0 software (GraphPad Software Inc).

## Supplementary Materials

The following are available online at https://www.mdpi.com/2073-4409/8/11/1359/s1, Figure S1: Behavioral assessment of MPTP vs non-MPTP treated mice. Figure S2: Tyrosine hydroxylase levels in NPs treated animals.
