# Supplementary material for: Peripheral Delivery of Neural Precursor Cells Ameliorates Parkinson’s Disease-Associated Pathology
Source: Cells. 2019 Oct 30;8(11):1359. doi: 10.3390/cells8111359 (PMC6912680; doi:10.3390/cells8111359)
Supplement: Supplementary file 1 [file cells-08-01359-s001.pdf]

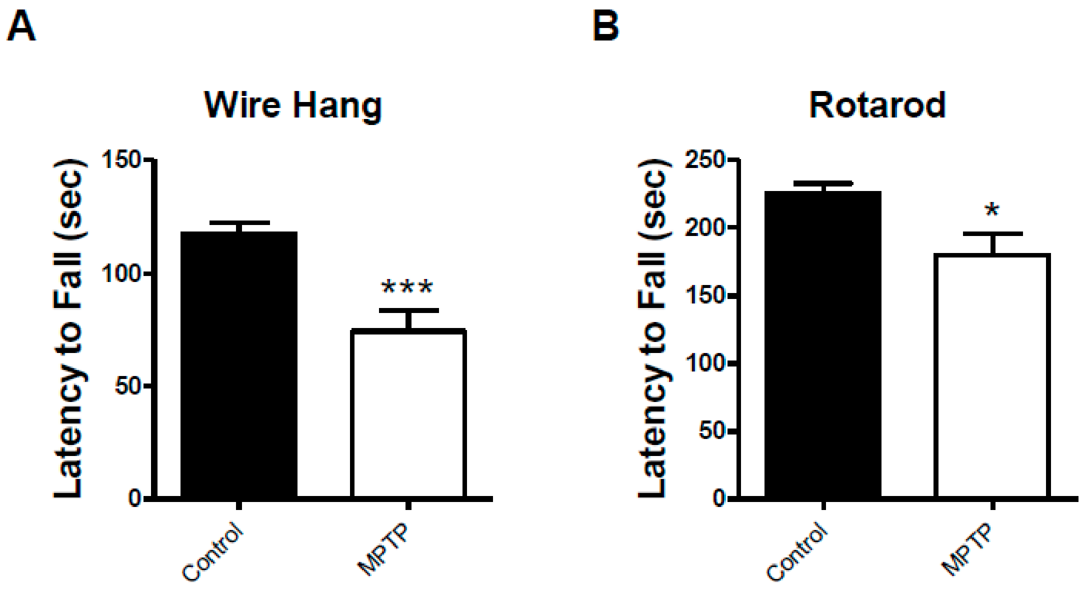

Figure S1. Behavioral assessment of MPTP vs non-MPTP treated mice. The bar graph represents the wire hang (A) and rotarod (B) assayed between MPTP-induced and non-MPTP induced mice at 10-25 days. Wire hang and rotarod latency to fall was significantly different in PD compared to non-PD mice. Data are expressed as mean ± s.e.m. Student t-test *p<0.05, ***p<0.001. N=10 mice/group.

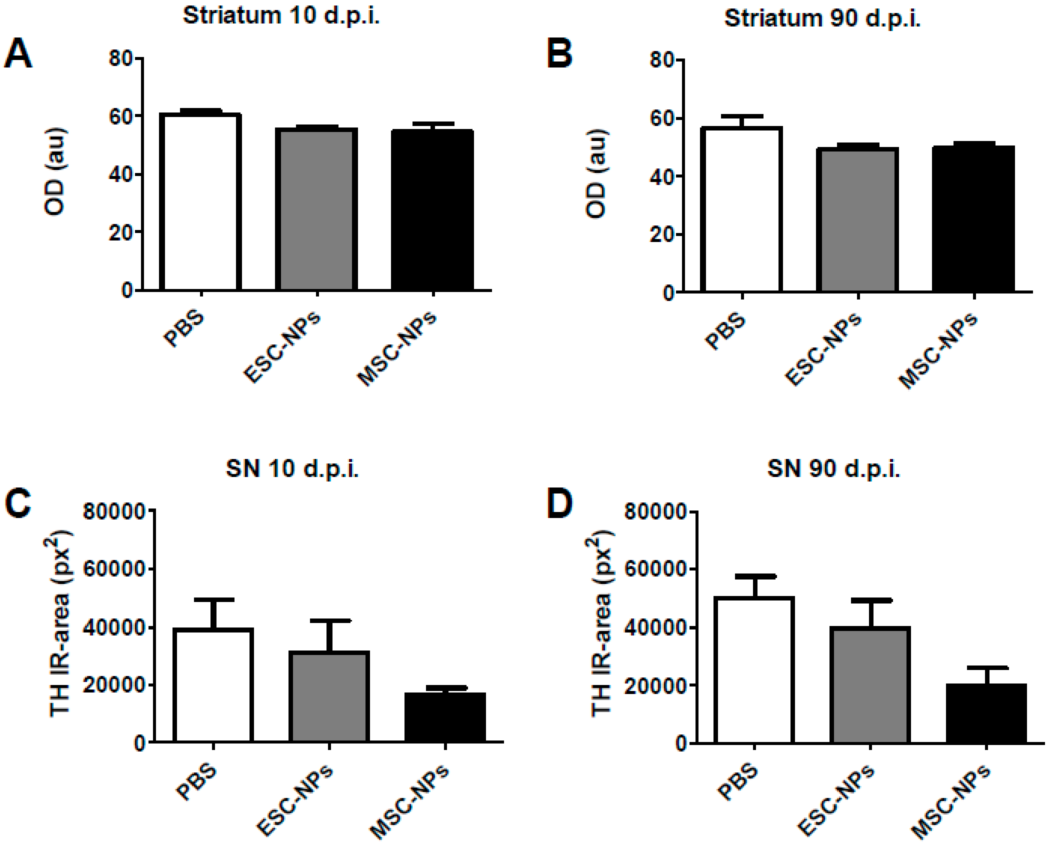

Figure S2. Tyrosine hydroxylase levels in NPs treated animals. Brain of experimental and control mice were histologically processed and tyrosine hydroxylase (TH) burden analyzed by immunohistochemistry. TH optical density (OD) was determine in the striatum 10 (A) and 90 (B) days post inoculation (d.p.i.). TH immunoreactive (IR) area (px^2) was quantified in the substantia nigra (SN) at 10 (C) and 90 (D) d.p.i. in PBS, ESC-NPs, and MSC-NPs treated animals. A-B: One-way ANOVA $p>0.05$, C: One-way ANOVA $p>0.05$, D: One-way ANOVA $p=0.048$, Bonferroni´s multiple comparison analysis $p>0.05$. N=6 animals/group, 8 (Striatum) 4 (SN) sections/animal.
